# Biomarkers of phosphate homeostasis and metabolic bone disease of prematurity: impact of early nutrition and regulation by vitamin D, iPTH, PTHrP, and FGF23

**DOI:** 10.1186/s12887-026-06940-1

**Published:** 2026-04-28

**Authors:** Shanshan Wu, Huifeng Zhang

**Affiliations:** 1 Department of Neonatology, the Second Hospital of Hebei Medical University, Hebei Key Laboratory of Early Life Health Promotion (SZX202419), Hebei Medical University, Xinhua District, Shijiazhuang City, Hebei Province China; 2 Department of Pediatrics, the Second Hospital of Hebei Medical University, Hebei Key Laboratory of Early Life Health Promotion (SZX202419), Hebei Medical University, Xinhua District, Shijiazhuang City, Hebei Province China

**Keywords:** Phosphate, Metabolic bone disease of prematurity, Calcium, iPTH, PTHrP, FGF23, Vitamin D, Nutrition

## Abstract

**Background:**

Global practices for supplementing calcium, phosphate, and vitamin D in early life, as well as for screening for metabolic bone disease of prematurity (MBDP), remain inconsistent. Furthermore, researchers have not yet established gestational age (GA)-specific reference intervals for phosphate homeostasis biomarkers. This study aimed to assess phosphate homeostasis parameters and MBDP risk factors under local neonatal nutritional practices and to characterize the serial levels of key hormones involved in phosphate metabolism, including 1,25-dihydroxyvitamin D (1,25(OH)_2_D), intact parathyroid hormone (iPTH), parathyroid hormone-related protein (PTHrP), and fibroblast growth factor-23 (FGF23).

**Methods:**

We conducted a prospective cohort study in 657 preterm infants with GAs of 26–36^+ 6^ weeks. We analysed serum phosphate (sP), calcium (sCa), and alkaline phosphatase (ALP) levels across different GAs and postnatal ages, and we identified risk factors for MBDP using univariate and multivariate logistic regression analyses. In a separate, in-depth cohort of 63 preterm infants, we measured and analysed urinary phosphate (uP), calcium (uCa), tubular reabsorption of phosphate (TRP), renal phosphate threshold concentration (Tp/GFR), and these hormones, and we assessed correlations using Pearson’s test.

**Results:**

Extremely preterm infants (EPIs) and very preterm infants (VPIs) developed low sP (median nadir: 1.54 mmol/L; interquartile range, IQR: 1.21–2.03), low sCa (nadir: 2.15 mmol/L; IQR: 2.07–2.22), and hypophosphaturia (uP nadir: 0.71 mmol/L; IQR: 0.31–1.66), along with elevated uCa (peak: 1.46 mmol/L; IQR: 1.18–1.70) and secondary hyperparathyroidism (peak iPTH: 29.2 pmol/L), which reflects insufficient early-life calcium-phosphate (Ca-P) intake. We observed lower sP levels (1.27 ± 0.20 mmol/L) in infants who started extra vitamin D supplementation after 14 postnatal days and in those with prolonged parenteral nutrition (PN) exposure. Our analysis showed a positive correlation between Tp/GFR and GA. We found no GA-based differences in the measured hormone levels. PTHrP levels progressively declined from umbilical cord blood (10.9 ± 2.4 pmol/L) to postnatal day 14 (9.1 ± 2.0 pmol/L). In contrast, initially very low iPTH levels (median 1.9 pmol/L) increased sharply after birth (median 28.3 pmol/L). FGF23 levels remained elevated throughout the study period.

**Conclusions:**

Our study reveals suboptimal Ca intake from PN, insufficient Ca-P supply via enteral nutrition (EN), and delayed vitamin D supplementation in the current local early-life nutrition protocol. The high prevalence of maternal vitamin D deficiency likely contributed to the vitamin D insufficiency that we observed in most preterm infants. Clinicians should utilize biomarkers including sP, sCa, uP, uCa, and iPTH to assess phosphate homeostasis and guide mineral supplementation in early life to reduce the incidence of MBDP.

## Introduction

Phosphate homeostasis imbalance is a key pathological feature of metabolic bone disease of prematurity (MBDP), primarily characterized by reduced bone mineral content [1, 2]. Currently, there is no consensus on several critical issues: (1) gestational age (GA)-specific reference ranges for serum phosphate (sP) and urinary phosphate (uP) in preterm infants; (2) whether the optimal phosphate supplementation dose should be GA-adjusted, as current international guidelines for parenteral [3] and enteral [4] nutrition provide standardized recommendations without explicit GA stratification; and (3) the identification of early predictive biomarkers for MBDP [5, 6]. These evidence gaps urgently require resolution. Furthermore, global practices for early-life calcium, phosphate, and vitamin D supplementation remain inconsistent [1].

Pediatric phosphate metabolism is regulated through intricate interactions among the intestines, bones, and kidneys, mediated primarily by 1,25-dihydroxyvitamin D (1,25(OH)_2_D), intact parathyroid hormone (iPTH), and fibroblast growth factor-23 (FGF23) [7]. In contrast, fetal sP maintenance depends on parathyroid hormone-related protein (PTHrP) and iPTH but not FGF23 or 1,25(OH)_2_D. This is evidenced by the classic hypoparathyroid-like phenotype, characterized by hypocalcemia and hyperphosphatemia, observed in PTHrP(–/–) animal models, confirming its essential role in fetal phosphate homeostasis [7]. Following premature birth, the abrupt cessation of placental calcium-phosphate (Ca-P) transfer triggers adaptive mechanisms, including an initial blunted iPTH response followed by a marked surge [7]. This transition involves (1) a shift from passive intestinal Ca-P absorption to active 1,25(OH)_2_D-dependent transport and (2) developmental adaptations in renal phosphate handling regulated by iPTH and FGF23 [7, 8]. However, reference ranges for these hormones regulating phosphate metabolism remain poorly established.

Based on these evidence gaps, we hypothesized that the current early-life nutritional protocol would be associated with a high prevalence of biochemical phosphate deficiency, particularly in extremely preterm infants (EPIs), and that this would be reflected in dynamic changes of phosphate-regulating hormones and urinary mineral excretion.

To test this hypothesis, the present study had two main objectives. First, we aimed to characterize the longitudinal profiles of serum phosphate, calcium, and alkaline phosphatase under the local nutritional protocol, and to identify the incidence and risk factors for MBDP. Second, we aimed to delineate the physiological adaptations in urinary mineral excretion and the serial profiles of key phosphate-regulating hormones, and to evaluate their potential as early biomarkers of phosphate homeostasis and MBDP.

## Materials and methods

### Study design and subjects

We conducted this prospective cohort study in the neonatal intensive care unit (NICU) of the Second Hospital of Hebei Medical University, a regional tertiary referral center. The study was approved by the hospital’s Research Ethics Committee. The eligibility criterion was infants with a GA of 26–36^+ 6^ weeks. We excluded infants with: (1) congenital malformations or genetic/metabolic diseases; (2) renal or congenital parathyroid disorders; (3) maternal parathyroid disease, renal disease, osteoporosis, or Ca-P abnormalities; or (4) incomplete clinical data. A significant proportion of the cohort were small-for-gestational-age (SGA) and experienced prolonged feeding intolerance, leading to extended dependence on parenteral nutrition (PN). Thus, PN was the primary determinant of total mineral intake during the early postnatal period. The study comprised two phases: Stage I (a large prospective cohort) and Stage II (a separate, in-depth cohort) for more intensive physiological investigation.

### Nutrition program

A standardized nutritional protocol was followed in our NICU. Infants received a combination of PN and enteral nutrition (EN), with the total fluid intake (PN + EN) starting at 60–80 mL/kg/day and advanced by 15–20 mL/kg/day to a target of 140–160 mL/kg/day.

EN was commenced within 24–48 h postnatally. Feedings were advanced daily by 1–2 mL/kg per feed (approximately 15–20 mL/kg/day). The feeding hierarchy prioritized mother’s own milk when available; in its absence, preterm formula (Nestlé) was used. Expressed breast milk was fortified with a human milk fortifier (Nestlé) when feeding volumes reached 80–100 mL/kg/day. Fortification was initiated at a ratio of 50 mL breast milk to 1 g of fortifier and increased to a ratio of 25 mL:1 g if well tolerated.

PN was initiated within 24 h of birth for infants weighing < 2 kg. Solutions were compounded by the Pharmacy Intravenous Admixture Service. Calcium was provided as calcium gluconate (10 mL: 2.25 mmol Ca²⁺) at a dose of 0.5–1 mmol/kg/day. Phosphate was provided as sodium glycerophosphate (1 mL: 1 mmol P³⁻) at a dose of 1 mmol/kg/day. The PN solution included a lipid emulsion and fat-soluble vitamins, providing a supplemental dose of approximately 50 IU/kg/day of vitamin D. Due to compatibility constraints (including the risk of calcium-phosphate precipitation in PN solutions [9] and the avoidance of calcium in peripheral lines), the potential to increase calcium delivery via PN was limited. Peripherally inserted central catheters (PICCs) were typically placed by the third postnatal day for infants weighing < 1.5 kg to facilitate mineral delivery.

Transition from PN to EN: As EN volumes increased, PN volumes were proportionally decreased. The intakes of calcium and phosphate from PN were empirically reduced during this transition phase. PN was discontinued entirely when full enteral feeding was achieved, defined as an intake of 120 mL/kg/day.

Vitamin D Supplementation: Given the low levels of vitamin D from PN and the variable content in unfortified breast milk, we administered a supplemental dose of 900 IU/day of vitamin D₃ orally, starting between 7 and 14 postnatal days. This was delivered as two separate commercial preparations: one providing 500 IU (Shandong Dain) and another providing 400 IU (Shandong Dain), in accordance with our unit’s protocol to ensure a consistent and sufficient daily dose.

The target nutrient intakes, as per the European Society for Paediatric Gastroenterology, Hepatology and Nutrition (ESPGHAN) recommendations against which our protocol was evaluated, were as follows: for PN, a calcium intake of 1.6–3.5 mmol/kg/day and a phosphate intake of 1.6–3.5 mmol/kg/day; for EN, a calcium intake of 3–5 mmol/kg/day and a phosphate intake of 2.2–3.7 mmol/kg/day [3, 4].

### Definitions of biochemical abnormalities

For the purpose of this study, hypophosphatemia was defined as a sP level < 1.8 mmol/L, a threshold consistently linked to the pathophysiology of MBDP. Hypocalcemia was defined as an albumin-adjusted sCa level < 2.2 mmol/L, which represents the lower limit of the normal reference range in our institutional laboratory. These thresholds were directly aligned with our clinical protocol, in which additional intravenous phosphate or calcium (0.5–1 mmol/kg/dose) was administered when serum levels fell below these limits.

MBDP was defined as the co-occurrence of a serum ALP activity > 500 U/L and hypophosphatemia (sP < 1.8 mmol/L) [10].

Hypophosphaturia and hypercalciuria were defined as a uP concentration < 1.0 mmol/L and a uCa concentration > 1.0 mmol/L, respectively. These thresholds are informed by the concept of slight surplus supply, which posits that optimal mineral status is associated with uP and uCa levels of 1–2 mmol/L [11].

### Biochemical parameters of phosphate homeostasis

For all infants in Stage I, blood samples were collected at birth (within 30 min of life) and then weekly until hospital discharge. Maternal serum 25(OH)D levels were measured in a subset of 306 mothers of the Stage I cohort using blood samples collected within 24 h prior to delivery. These mothers were consecutively enrolled during the same study period; no additional selection criteria were applied. In the Stage II separate, in-depth cohort, additional blood and spot urine samples were collected at birth and weekly. Following sample collection and centrifugation, the supernatants were stored at -80 °C until analysis. Biochemical parameters—including sP, sCa, ALP, serum creatinine (sCr), uP, uCa, and urine creatinine (uCr) in spot urine samples—were measured using a fully automated biochemical analyser. The albumin-adjusted calcium level was calculated as total calcium (mmol/L) + 0.012 × (39.9 - albumin [g/L]). This adjustment was employed to provide a better estimate of physiologically active calcium, given the high prevalence of hypoalbuminemia in our preterm cohort, despite the recognized lack of established reference intervals for adjusted calcium in this population [12]. Serum levels of PTHrP, iPTH, 25(OH)D, and FGF23 were quantified by enzyme-linked immunosorbent assay (ELISA; Shanghai Ruifan Biological Technology Co., Ltd.). The iPTH assay had a measuring range of 2.5–80 pmol/L, with a manufacturer-reported normal reference range of 1.6–6.9 pmol/L for adults and a sensitivity < 1.0 pmol/L. It is important to note that validated, GA-specific reference intervals for iPTH in preterm infants are not currently available. TRP was calculated as follows: 1 - (uP × sCr)/(uCr × sP). The renal phosphate threshold concentration (Tp/GFR) was determined as sP - (sCr × uP/uCr). For cases with TRP > 0.86, Tp/GFR was calculated using the correction factor: α × sP, where α = (0.3 × TRP)/(1–0.8 × TRP), accounting for the curvilinear relationship between sP and TRP [13].

### Statistical analyses

We performed all statistical analyses using IBM SPSS Statistics version 27.0.1. We assessed data distribution using the Shapiro-Wilk test and verified homogeneity of variances using Levene’s test. We present normally distributed data as mean ± standard deviation (SD) and non-normally distributed data as median with interquartile range (IQR). We compared continuous variables across GA groups or other clinical categories using the following approach: if data satisfied both normality and homogeneity of variances, we employed one-way analysis of variance (ANOVA), followed by Fisher’s Least Significant Difference (LSD) test for post-hoc pairwise comparisons. If the assumptions were violated, we used the non-parametric Kruskal-Wallis H test, followed by Bonferroni-corrected Mann-Whitney U tests for post-hoc pairwise comparisons. We compared categorical variables using the Chi-square test or Fisher’s exact test, as appropriate. We employed multiple linear regression with sP as the dependent variable, incorporating perinatal characteristics, neonatal complications, feeding types, and other potential Ca-P balance modifiers as independent variables. We performed logistic regression analysis with MBDP as the outcome variable. We assessed correlations between Ca-P-related biochemical parameters using Pearson’s tests. We considered a two-tailed p-value < 0.05 as statistically significant.

## Results

### Study cohort

A total of 695 preterm infants with GAs of 26–36^+ 6^ weeks were initially screened. We excluded 38 infants due to: (1) congenital malformations or genetic/metabolic diseases; (2) renal or congenital parathyroid disorders; (3) maternal parathyroid disease, renal disease, osteoporosis, or Ca-P abnormalities; or (4) incomplete clinical data. The final analysis included 657 infants in Stage I (enrolled from June 1, 2022, to May 31, 2023). A subsequent separate, in-depth cohort of 63 infants was enrolled from June 1 to June 30, 2023, for more intensive physiological investigation.

### Patient characteristics and biochemical parameters in Stage I

**Fig. 1:**
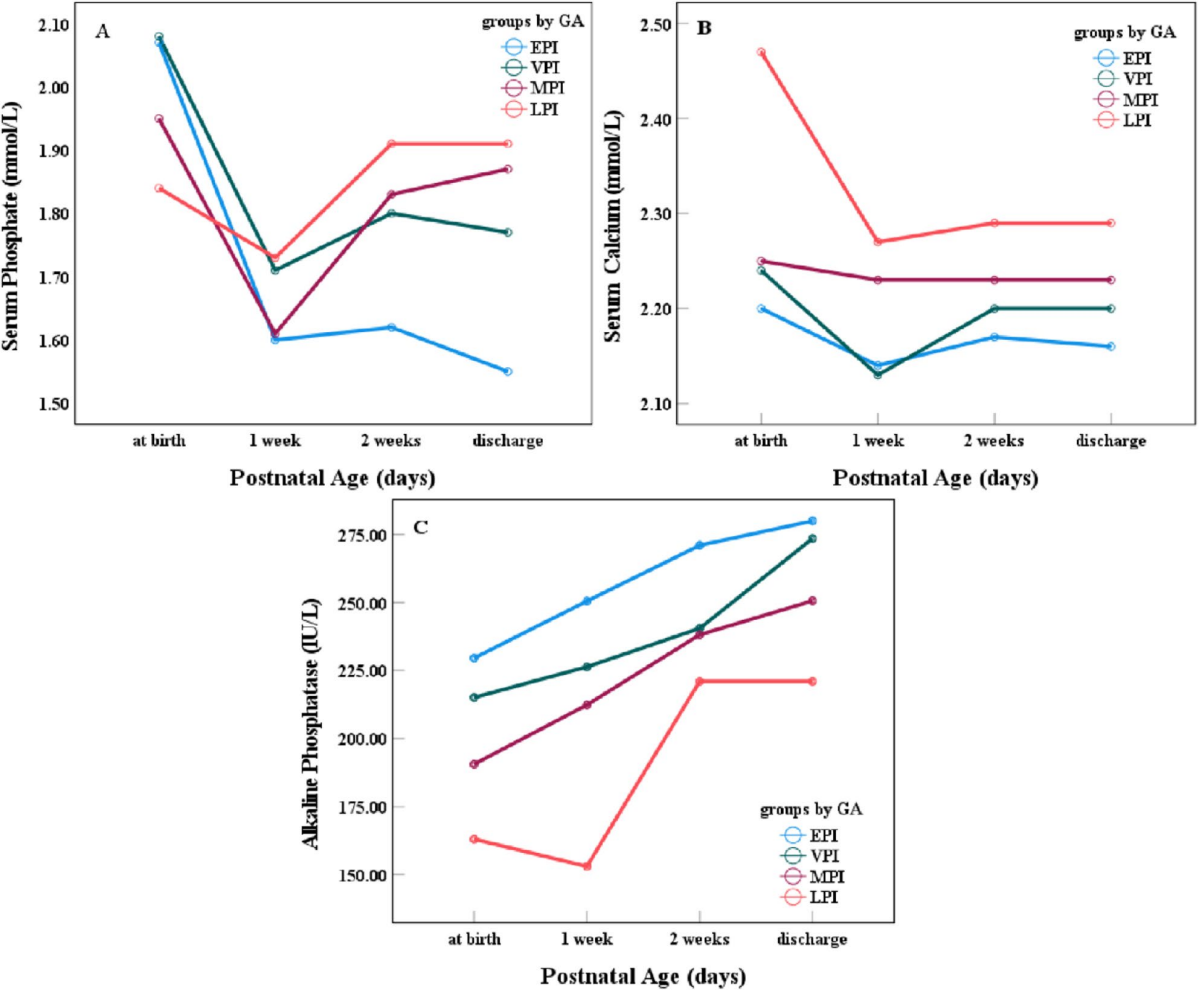
Postnatal trends in serum biochemical parameters across gestational age (GA) groups. **A** Serum phosphate (sP) levels. **B** Serum calcium (sCa) levels. **C** Alkaline phosphatase (ALP) levels. In all panels, the X-axis represents postnatal age. Lines represent median values for the following GA groups: extremely preterm infant (EPI, < 28 weeks), very preterm infant (VPI, 28–31^+ 6^ weeks), moderate preterm infant (MPI, 32–33^+ 6^ weeks), and late preterm infant (LPI, ≥ 34 weeks)

The perinatal, clinical, and nutritional characteristics of the 657 preterm infants are summarized in Table 1–3. A total of 657 preterm infants were included in Stage I. The median GA was 29.9 weeks (IQR: 28.6–31.2), and the median BW was 1480 g (IQR: 1150–1750). Among them, 21.5% were SGA, and 11.3% were diagnosed with MBDP. Table 1 outlines the baseline demographics of a high-risk cohort, characterized by a high prevalence of factors such as SGA status and maternal comorbidities. Compared with the current ESPGHAN recommendations for parenteral [3] and enteral [4] nutrition, the observed nutrient intake in our local early-life nutrition program showed deficiencies in calcium intake from PN, inadequate Ca-P intake from EN, and a comparable calcium-to-phosphate ratio (Ca: P) (Table 2). This documented inadequacy in mineral supply serves as the pivotal exposure underlying the subsequent biochemical abnormalities. Comparative analysis revealed significant differences across GA groups: (1) Levels of sP were inversely correlated with GA at birth (*p* < 0.001). (2) Biochemical evidence of mineral deficiency was most pronounced in the most premature infants. As detailed in Table 3 and visualized in Fig. 1A and B, the median sP and sCa levels in EPIs and very preterm infants (VPIs) persistently remained below the thresholds for hypophosphatemia (< 1.8 mmol/L) and hypocalcemia (< 2.2 mmol/L) throughout the first weeks of life and at hospital discharge. In contrast, median levels in moderate preterm infants (MPIs) and late preterm infants (LPIs) were consistently at or above these thresholds. The fact that the median values for EPIs and VPIs were sustained below these clinical and laboratory cut-offs strongly indicates a high prevalence of these biochemical abnormalities within these groups, confirming their heightened risk for MBDP. (3) ALP levels were significantly elevated in the EPI and VPI groups from birth to discharge compared with those in the MPI and LPI groups (280.0 vs. 252.5 U/L, *p* < 0.001) (Table 3).

**Table 1:** Stage I preterm infants (*n* = 657): perinatal and clinical characteristics

		Count	Frequency (%)
Gender	Male	330	50.2
	Female	327	49.8
GA	< 28w	36	5.5
	28–31^+ 6^w	419	63.8
	32–33^+ 6^w	178	27.1
	34–36^+ 6^w	24	3.7
BW	< 1Kg	71	10.8
	1–1.49Kg	334	50.8
	1.5–2.49Kg	248	37.7
	≥ 2.5Kg	4	0.6
Perinatal characteristics	SGA	141	21.5
	Twins	142	21.6
	Cesarean section	594	90.4
	Pregnancy-induced hypertension	345	52.5
	Gestational diabetes mellitus	158	24.0
	Placenta previa	114	17.4
types of feeding	Breastmilk	176	26.8
	Infant formula	320	48.7
	Mixed	161	24.5
Morbidity	NRDS	443	67.4
	NEC	10	1.5
	Sepsis	63	9.6
	Cholestasis	19	2.9
	BPD	135	20.5

*GA* gestational age, *BW* birth weight, *SGA* small for gestational age, defined as birth weight below the 10th percentile for gestational age according to the Fenton growth charts, *NRDS* neonatal respiratory distress syndrome (respiratory distress + oxygen requirement + characteristic chest X-ray findings), *NEC* necrotizing enterocolitis (Bell staging criteria), *sepsis* positive blood culture within 7 postnatal days, *BPD* bronchopulmonary dysplasia (GA-based diagnosis at 36 weeks postmenstrual age)

**Table 2:** Observed early-life nutrient intake (Mean and 95% Confidence Interval) in the study cohort

Nutrient Intake	Postnatal Week 1	Postnatal Week 2	Postnatal Week 3	Postnatal Week 4
Calcium intake, PN + EN (mmol/kg/day)	0.8(0.3–1.2)	1.8(1.5–2.0)	2.6(2.4–2.8)	2.6(2.4–2.8)
Phosphate intake, PN + EN (mmol/kg/day)	1.0(0.3–1.7)	2.1(1.9–2.2)	2.6(2.5–2.8)	3.2(3.0–3.3)
Ca: P intake molar ratio	1.0(0.6–1.4)	0.9(0.8–0.9)	1.0(1.0–1.0)	1.1(1.1–1.1)

Reference: The observed intakes are compared against the European Society for Paediatric Gastroenterology, Hepatology and Nutrition (ESPGHAN) recommendations for preterm infants: calcium 1.6–3.5 mmol/kg/day, phosphate 1.6–3.5 mmol/kg/day [3]

*Abbreviations*: *PN* parenteral nutrition, *EN* enteral nutrition; *Ca: P* calcium-to-phosphate molar ratio

**Table 3:** Comparative analysis of biochemical parameters across gestational age (GA) groups and postnatal age

	< 28w	28–31^+ 6^w	32–33^+ 6^w	34–36^+ 6^w	*p*-Value
sP at birth (mmol/L), median (IQR)	2.11 (1.89–2.26) *	2.07 (1.90–2.28) *	1.97 (1.77–2.13)	1.91 (1.59–2.06)	< 0.001 (F = 7.457)
sP at week 1 (mmol/L), median (IQR)	1.54 (1.21–2.03)	1.71 (1.42–2.01) *	1.58 (1.30–1.90)	1.69 (1.41–2.05)	0.049 (H = 7.878)
sP at week 2 (mmol/L), median (IQR)	1.57 (1.39–1.89) *	1.81 (1.61–1.99) *	1.83 (1.68–2.09)	NON	0.001 (H = 16.016)
sP hospital discharge (mmol/L), median (IQR)	1.55 (1.30–1.74) *	1.78 (1.60–1.94) *	1.87 (1.71–2.06)	NON	< 0.001 (H = 32.936)
sCa at birth (mmol/L), median (IQR)	2.17 (2.04–2.38)	2.23 (2.13–2.32)	2.26 (2.18–2.36)	2.46 (2.38–2.58) *	< 0.001 (F = 12.04)
sCa at week 1 (mmol/L), median (IQR)	2.15 (2.07–2.22) *	2.17 (2.06–2.26) *	2.22 (2.13–2.30)	2.26 (2.23–2.33)	< 0.001 (H = 25.128)
sCa at week 2 (mmol/L), median (IQR)	2.18 (2.08–2.30) *	2.21 (2.12–2.29) *	2.23 (2.15–2.33)	NON	< 0.001 (H = 19.964)
sCa hospital discharge (mmol/L), median (IQR)	2.16 (2.06–2.24) *	2.20 (2.11–2.28) *	2.22 (2.15–2.33)	NON	< 0.001 (H = 28.265)
ALP at birth (U/L), median (IQR)	233.5 (175.5–269.3) *	210.0 (173.0–249.8) *	182.0 (148.3–225.8)	163.0 (131.5–250.5)	< 0.001 (H = 23.001)
ALP at week 1 (U/L), median (IQR)	250.5 (199.5–343.5) *	215.0 (177.0–270.8) *	209.0 (167.3–241.3)	153.0 (122.5–216.5)	< 0.001 (H = 18.971)
ALP at week 2 (U/L), median (IQR)	271.0 (213.3–370.5) *	240.5 (201.3–305.8) *	232.0 (177.3–278.0)	NON	0.025 (H = 9.339)
ALP hospital discharge (U/L), median (IQR)	280.0 (250.0–527.3) *	273.5 (219.0–352.0) *	252.5 (194.3–286.8)	NON	< 0.001 (H = 24.198)

*Abbreviations*: *GA* gestational age, *sP* serum phosphate, *sCa* serum calcium, *ALP* alkaline phosphatase, *IQR* interquartile range

Data are presented as median (IQR). The p-Value is for the overall comparison across all GA groups. ‘H’ denotes the test statistic for the Kruskal-Wallis H test; ‘F’ denotes the test statistic for the one-way Analysis of Variance (ANOVA). Post-hoc pairwise comparisons were performed following a significant overall test

The asterisk (*) indicates a statistically significant difference (*p* < 0.05). ‘NON’ indicates that data were not available for that time point as infants in that GA group had typically been discharged

Figure 1A and B illustrate the week-by-week trends in sP and sCa, demonstrating a parallel decline. Both sP and sCa reached their nadir by the end of the first week. EPIs demonstrated the most significant decreases: median sP nadir: 1.54 mmol/L (IQR: 1.21–2.03); median sCa nadir: 2.15 mmol/L (IQR: 2.07–2.22). Although levels gradually increased thereafter, they remained significantly lower in the EPI and VPI groups than in the MPI and LPI groups throughout the study period. In contrast, ALP levels exhibited a sustained increase in the EPI and VPI groups (Fig. 1C).

Seventy-four of the 657 infants were diagnosed with MBDP (sP < 1.8 mmol/L and ALP > 500 U/L), a condition that typically occurs approximately 6 weeks after birth. The clinical characteristics of the patients are summarized in Table 4. Univariate analysis demonstrated that lower GA, lower birth weight (BW), SGA status, and delayed vitamin D_3_ supplementation initiation (> 14 days) were significant risk factors for MBDP. Additionally, MBDP infants had higher incidences of neonatal respiratory distress syndrome (NRDS), necrotizing enterocolitis (NEC), bronchopulmonary dysplasia (BPD), cholestasis, and sepsis than controls did. In contrast, preterm formula feeding emerged as a protective factor.

**Table 4:** Comparisons of the perinatal and clinical characteristics of metabolic bone disease of prematurity (MBDP) and non-MBDP patients

		MBDP	non-MBDP	*p*-Value
Gender	Male	40 (54.1%)	290 (49.7%)	0.485
	Female	34 (45.9%)	293 (50.3%)	
GA	< 28w	15 (20.3%) ^a^	21 (3.6%) ^a^	< 0.001
	28–31^+ 6^w	51 (68.9) ^b^	368 (63.1%) ^b^	
	32–33^+ 6^w	7 (9.5%) ^c^	171 (29.3%) ^c^	
	34–36^+ 6^w	1 (1.4%)	23 (3.9%)	
Perinatal characteristics	SGA	23 (31.1%)	118 (20.2%)	0.032
	Twins	14 (18.9%)	127 (21.8%)	0.572
	Cesarean section	58 (78.4%)	536 (91.9%)	< 0.001
	Pregnancy-induced hypertension	38 (51.4%)	307 (52.7%)	0.832
	Gestational diabetes mellitus	16 (21.6%)	142 (24.4%)	0.604
	Placenta previa	13 (17.6%)	101 (17.3%)	0.958
Types of feeding	Breastmilk	26 (35.1%) ^a^	150 (25.7%) ^a^	< 0.001
	Infant formula	13 (23.0%) ^b^	303 (52.0%) ^b^	
	Mixed	31 (41.9%) ^a^	130 (22.3%) ^a^	
Morbidity	NRDS	66 (89.2%)	377 (64.7%)	< 0.001
	NEC	4 (5.4%)	6 (1.0%)	0.017
	Sepsis	17 (23.0%)	46 (7.9%)	< 0.001
	Cholestasis	9 (12.2%)	10 (1.7%)	< 0.001
	BPD	36 (48.6%)	99 (17.0%)	< 0.001

*Abbreviations*: *GA* gestational age, *BW* birth weight, *SGA* small for gestational age, *NRDS* neonatal respiratory distress syndrome, *NEC* necrotizing enterocolitis, *BPD* bronchopulmonary dysplasia

The superscript letters (^a^, ^b^, ^c^) denote categories that are statistically significantly different from each other (*p* < 0.05) in the post-hoc analysis

Perinatal and clinical variables were analysed using logistic regression, with MBDP as the dependent variable (Table 5). The analysis revealed that delayed initiation of vitamin D_3_ supplementation (> 14 days; adjusted odds ratio (OR) = 5.121), NRDS (2.747), NEC (6.033), BPD (2.587), and prolonged PN (2.488) were risk factors, whereas low birth weight (LBW) (0.372 vs. extremely low birth weight (ELBW)) and preterm formula (0.42) were protective factors.

**Table 5:** Logistic regression analysis of risk factors for metabolic bone disease of prematurity (MBDP)

Risk Factor	OR	95% CI	*p*-Value
Model 1: Forward Stepwise Selection			
NRDS	2.75	1.23–6.13	0.014
NEC	6.03	1.46–24.98	0.013
BPD	2.59	1.48–4.51	0.001
Late initiation of vitamin D_3_ (> 14 days)	5.12	3.00–8.75	< 0.001
Model 2: Forced Entry of Preselected Variables			
NRDS	1.79	0.74–4.32	0.195
NEC	3.22	0.61–17.07	0.17
BPD	1.03	0.50–2.13	0.938
Late initiation of vitamin D_3_ (> 14 days)	5.85	3.24–10.55	< 0.001
LBW vs. ELBW	0.37	0.12–1.13	0.081
Preterm formula feeding	0.42	0.20–0.88	0.021
Prolonged parenteral nutrition (> 40 days)	2.49	0.93–6.64	0.069

Model Specification: The outcome variable for all analyses is the diagnosis of MBDP. Model 1 was constructed using forward stepwise likelihood ratio (LR) selection. Model 2 was constructed by forcing the entry of clinically preselected variables

*Abbreviations*: *NRDS* neonatal respiratory distress syndrome, *NEC* necrotizing enterocolitis, *BPD* bronchopulmonary dysplasia, *LBW* low birth weight, *ELBW* extremely low birth weight, *OR* odds ratio, *CI* confidence interval

Model Fit: Omnibus test, *p* < 0.001; Hosmer-Lemeshow test, *p* = 0.295; predictive accuracy = 89%

Multiple linear regression analysis (Table 6) was conducted to identify key determinants of sP levels, yielding the following predictive equation: sP (mmol/L) = 2.498 + 0.016 × x_1_ − 0.042 × x_2_ − 0.004 × x_3_ − 0.001 × x_4_ − 0.329 × x_5_ − 0.077 × x_6_. The model revealed a weak positive association between sP and GA, whereas weak inverse relationships were observed for the following sequentially entered variables: pregnancy-induced hypertension, time to achieve full enteral feeding, ALP levels, sCa levels, and delayed vitamin D_3_ supplementation initiation.

**Table 6:** Multiple linear regression: effects of clinical characteristics on serum phosphate (sP)

Independent Variables	B-Value	*p*-Value
Constant	2.498	< 0.001
GA	0.016	0.037
Pregnancy-induced hypertension	-0.042	0.063
Days of achieving total enteral feeding	-0.004	0.006
ALP	-0.001	< 0.001
sCa	-0.329	< 0.001
Days starting extra vitamin D_3_ supply	-0.077	0.005

Model Assumptions and Fit: Durbin-Watson statistic = 1.960; residuals were normally distributed with equal variance; no multicollinearity was detected (tolerance > 0.1)

*Abbreviations*: *sP* serum phosphate, *GA* gestational age, *sCa* serum calcium, *ALP* alkaline phosphatase

The model demonstrated significant fit: F = 34.419, *p* < 0.001, adjusted R² = 0.29

### Biochemical parameters in Stage II

**Fig. 2:**
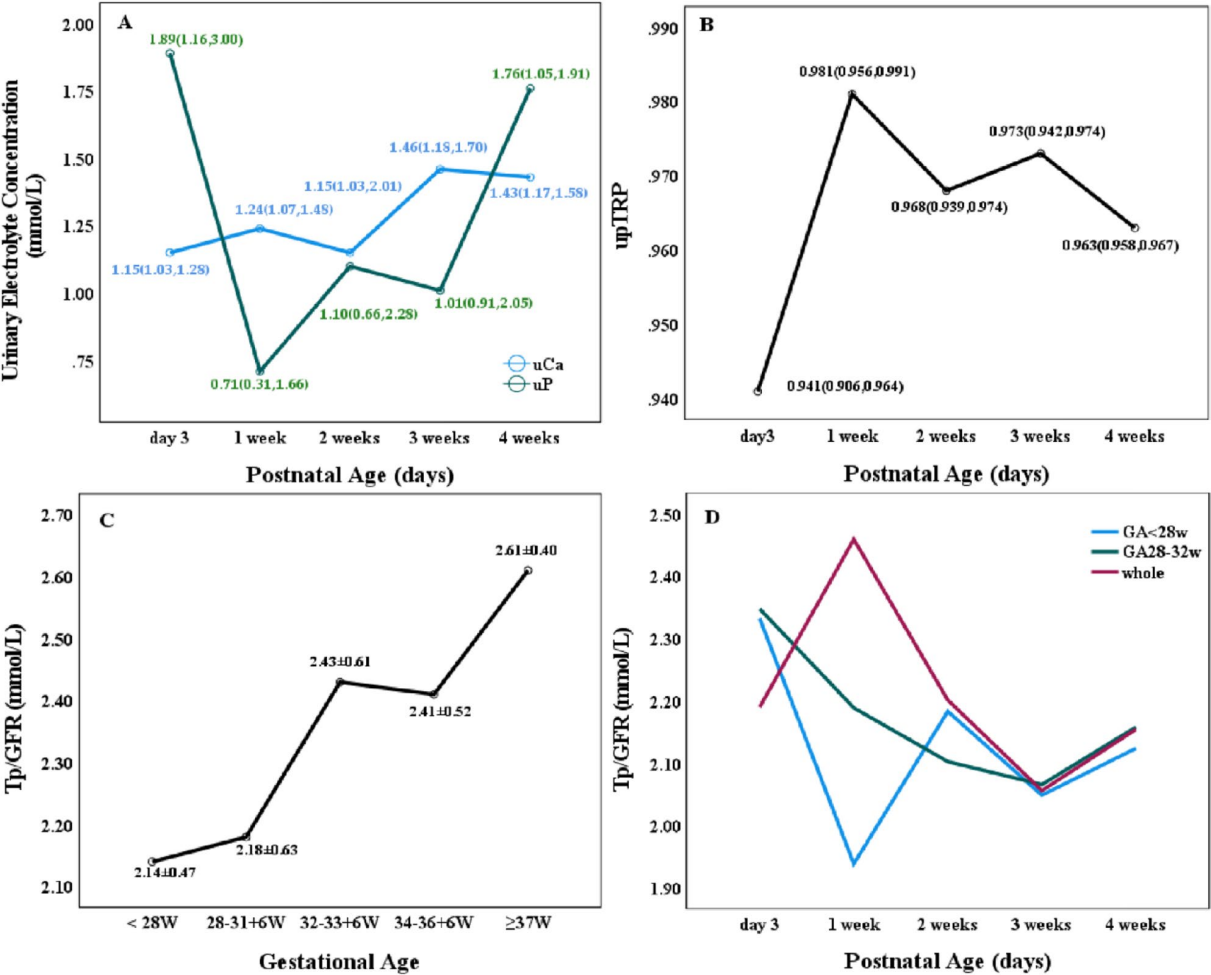
Postnatal trends in urinary mineral handling across gestational age (GA) and postnatal age. **A** Urinary calcium (uCa) and urinary phosphate (uP) levels. **B** Tubular reabsorption of phosphate (TRP). **C** Theoretical renal phosphate threshold (Tp/GFR) across GA groups. **D** Tp/GFR across postnatal age. Data points represent median values in (**a**) and (**b**), and mean values in (**c**) and (**d**). Lines connect the points to illustrate trends. In panel (**d**), the Tp/GFR of specific gestational age groups (< 28 weeks, blue; 28–32 weeks, green) are plotted against the overall study cohort (purple). Abbreviations: uCa, urinary calcium; uP, urinary phosphate; TRP, tubular reabsorption of phosphate; Tp/GFR, theoretical renal phosphate threshold; GA, gestational age

The postnatal trends in urinary parameters across the first four weeks of life are shown in Fig. 2A and D. Data are presented as weekly median values for the entire cohort (Figs. 2A, B, D) or by GA subgroup (Fig. 2C). All GA groups exhibited persistently elevated uCa excretion (Fig. 2A). The peak median uCa concentration reached 1.46 mmol/L (IQR 1.18–1.70). Throughout the weekly sampling period, median uCa levels consistently exceeded the 1.0 mmol/L threshold, a cut-off value established in prior studies using bone mineral content as an endpoint [11, 14]. In contrast, uP levels decreased significantly after birth, reaching a nadir in the second week before rebounding, but remained below the birth value (0.71 vs. 1.89 mmol/L) (Fig. 2A). Concurrently, TRP was initially high (> 85%) and demonstrated a postnatal age-dependent increase, stabilizing above 95% within four weeks (Fig. 2B). Analysis of Tp/GFR by GA group (Fig. 2C) revealed a positive correlation with maturity. The lowest values were observed in EPIs (2.14 ± 0.47 mmol/L), with progressively higher values in MPIs (2.43 ± 0.61 mmol/L), reaching peak levels in term infants (2.61 ± 0.40 mmol/L). While Tp/GFR in the overall cohort increased from birth, peaked at one week, and then gradually declined, infants with a GA < 32 weeks presented three distinct patterns: (1) absence of an early peak; (2) a delayed, slow increase emerging near week 4 (remaining below birth baseline values); and (3) higher values in the 28–32-week GA subgroup than in the ≤ 28-week GA infants (Fig. 2D).

**Fig. 3:**
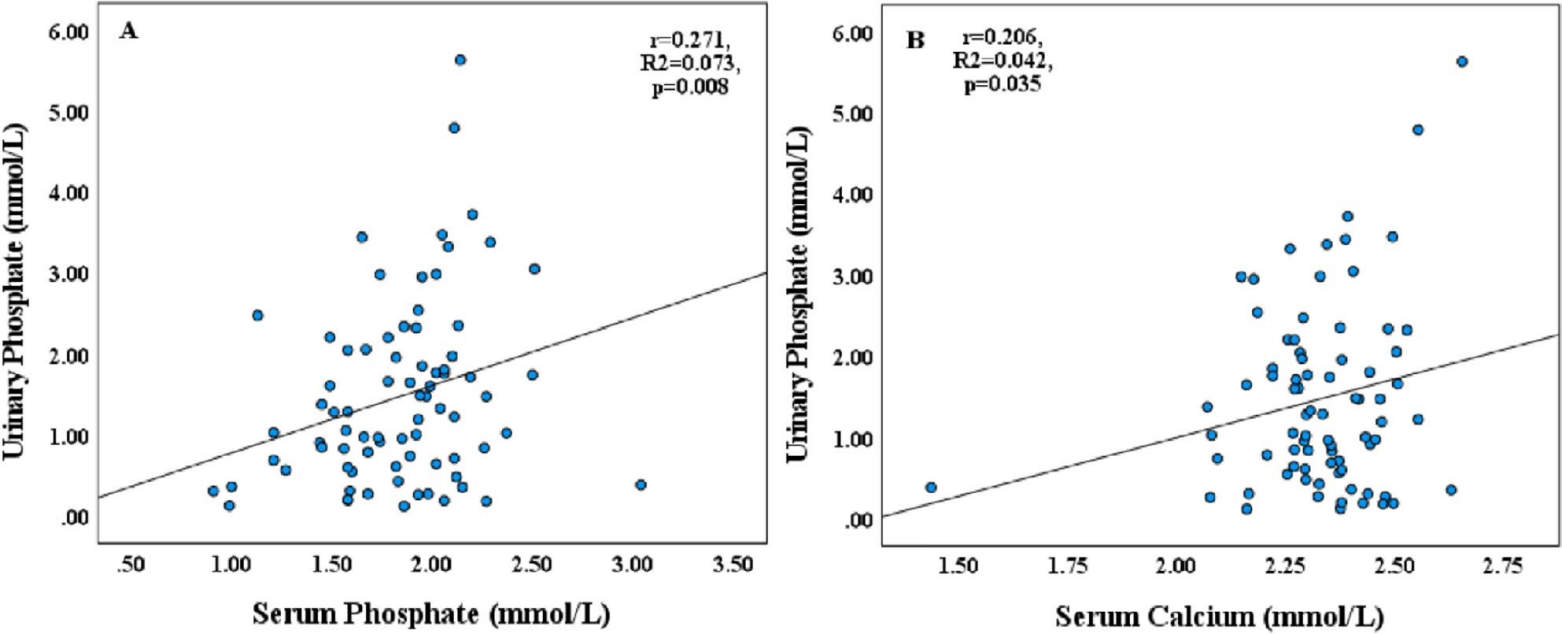
Linear regression analyses of urinary and serum biochemical parameters. **A** Scatter plot of urinary phosphate levels against serum phosphate levels. **B** Scatter plot of urinary phosphate levels against serum calcium levels. Solid lines represent the line of best fit from linear regression analysis. The Pearson correlation coefficient (*r*) and the corresponding *p*-value are indicated within each panel. hormones regulating phosphate metabolism. This short sentence serves as a subheading for the following content

Linear regression analysis revealed weak positive correlations between uP and both sP and sCa (Fig. 3A and B). In contrast, we observed no significant associations between uCa and either sCa or sP.

Pre-delivery analysis of 306 maternal serum 25(OH)D levels revealed widespread deficiency (median: < 25–27.5 nmol/L). The results of the Kruskal‒Wallis test (H = 1.954, *p* = 0.582) confirmed that there was no GA-dependent variation (Table 7).

**Table 7:** Hormone profiles regulating phosphate metabolism in preterm infants across gestational age (GA) and postnatal age (*n* = 63)

	< 28 W		28–31^+ 6^W	32 W-33^+ 6^W	34 W-36^+ 6^W	≥ 37 W	*p*-Value
25(OH)D of cord blood (nmol/L), mean ± SD	59.5 ± 16.3		71.3 ± 13.0	79.5 ± 14.5	70.5 ± 18.0	85.5 ± 14.8	0.155 (F = 1.770)
25(OH)D at birth (nmol/L), mean ± SD	65.5 ± 16.8		89.3 ± 16.8	72.3 ± 19.5	68.8 ± 17.3	NON	0.2 (F = 1.672)
25(OH)D at the end of week 1(nmol/L), mean ± SD	NON		87.0 ± 15.0	72.3 ± 27.8	78.0 ± 18.0	NON	0.39 (F = 1.116)
25(OH)D at the end of week 2(nmol/L), mean ± SD	NON		74.0 ± 9.8	89.3 ± 14.8	NON	NON	0.542 (F = 0.868)
iPTH of cord blood (pmol/L), median (IQR)	2.1 (1.9–2.3)		1.9 (1.5–,2.6)	1.4 (1.4–1.6)	1.9 (1.5–2.2)	2.1 (2.0–2.5)	0.140 (H = 6.921)
iPTH at birth (pmol/L), median (IQR)	25.8 (24.5–26.7)		29.2 (28.7–31.2)	26.8 (25.9–29.2)	27.1 (18.9–31.8)	NON	0.596 (H = 2.777)
iPTH at the end of week 1 (pmol/L), median (IQR)	NON		23.7 (22.6–23.7)	21.8 (17.2–28.0)	27.2 (22.6–29.9)	NON	0.832 (H = 1.471)
iPTH at the end of week 2 (pmol/L), median (IQR)	NON		28.2 (22.9–28.4)	22.4 (18.1–27.1)	NON	NON	0.446 (H = 2.664)
PTHrP of cord blood (pmol/L), mean ± SD	9.1 ± 1.3		11.8 ± 1.9	9.0 ± 2.4 *	11.4 ± 2.6	11.0 ± 2.3	0.05 (F = 2.607)
PTHrP at birth (pmol/L), mean ± SD	11.6 ± 0.2		10.7 ± 2.0	12.3 ± 1.4	11.4 ± 2.4	11.0 ± 2.3	0.427 (H = 3.846)
PTHrP at the end of week 1 (pmol/L), mean ± SD	NON		10.0 ± 2.5	9.8 ± 2.8	12.2 ± 2.7	NON	0.345 (F = 1.174)
PTHrP at the end of week 2 (pmol/L), mean ± SD	NON		8.5 ± 2.0	9.7 ± 2.8	NON	NON	0.918 (F = 0.216)
FGF23 of cord blood (pg/ml), mean ± SD	166.6 ± 8.6		197.3 ± 45.0	193.3 ± 44.5	188.7 ± 33.6	173.0 ± 29.4	0.699 (F = 0.551)
FGF23 at birth (pg/ml), mean ± SD	148.8 ± 24.1		215.9 ± 51.3	169.6 ± 38.4	187.1 ± 45.8	215.0 ± 31.6	0.279 (F = 1.371)
FGF23 at the end of week 1 (pg/ml), mean ± SD	NON		175.4 ± 32.0	215.1 ± 33.8	196.3 ± 45.5	NON	0.329 (F = 1.233)
FGF23 at the end of week 2 (pg/ml), mean ± SD	NON		221.1 ± 49.9	211.4 ± 33.7	NON	NON	0.467 (F = 1.048)
Maternal serum 25(OH)D(nmol/L), median (IQR)	37.0 (19.0–46.8)	29.0 (21.0–38.3)		29.8 (22.5–45.5)	29.3 (20.8–41.0)	NON	0.582 (H = 1.954)

Data Presentation and Statistics: Data are presented as mean ± SD or median (IQR), as appropriate. The p-Value is for the overall comparison across all GA groups. ‘H’ denotes the test statistic for the Kruskal-Wallis H test; ‘F’ denotes the test statistic for the one-way Analysis of Variance (ANOVA). Post-hoc pairwise comparisons were performed following a significant overall test

*Abbreviations*: *GA* gestational age, *25(OH)D* 25-hydroxyvitamin D, *SD* standard deviation, *iPTH* intact parathyroid hormone, *IQR* interquartile range, *PTHrP* parathyroid hormone-related peptide, *FGF23* fibroblast growth factor-23

The asterisk (*) indicates a significant difference (*p* < 0.05) in the post-hoc analysis. ‘NON’ indicates that data were not available for that time point

**Fig. 4:**
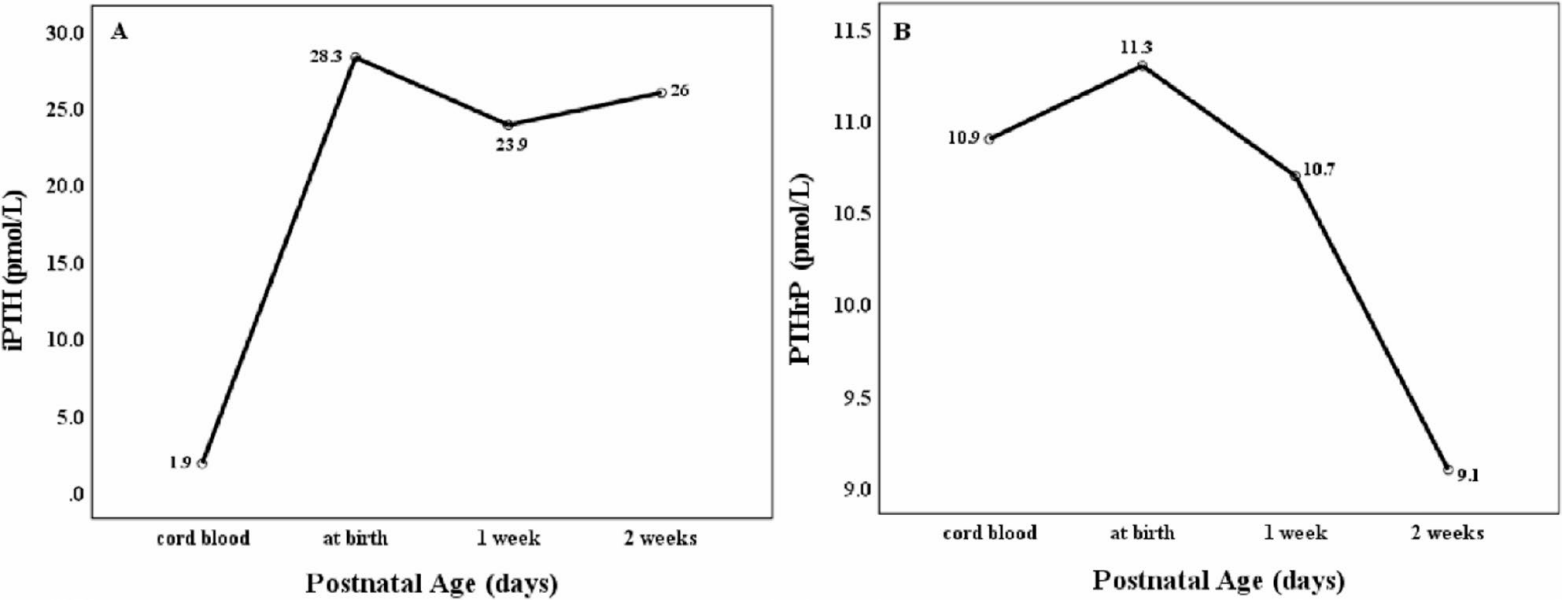
Postnatal changes in parathyroid hormone and related peptide levels. **A** Serum levels of intact parathyroid hormone (iPTH). **B** Serum levels of parathyroid hormone-related peptide (PTHrP). Data points in (**a**) represent median values; data points in (**b**) represent mean values. Lines connect the points to illustrate postnatal trends

We analysed 90 serum samples from 63 preterm infants and found distinct patterns in hormone regulating phosphate metabolism levels. Serum 25(OH)D concentrations showed no significant GA-dependent differences in either cord blood or postnatal measurements, although a nonsignificant increasing trend with advancing postnatal age was noted (from 73.8 ± 15.7 to 84.9 ± 12.6 nmol/L; ANOVA: F = 1.221, *p* = 0.307). FGF23 levels did not establish consistent GA-related or postnatal age-dependent patterns, with high ranges remaining unchanged (from 189.8 ± 38.8 to 199.9 ± 41.4 pg/ml). Strikingly, cord blood exhibited profoundly lower iPTH levels (median 1.9 pmol/L) than postnatal values (median 28.3 pmol/L; Kruskal‒Wallis test: H = 68.811, *p* < 0.001), although no GA-specific variations were observed. In contrast, PTHrP showed a significant postnatal decline (from 10.9 ± 2.4 pmol/L to 9.1 ± 2.0 pmol/L; *p* < 0.05), revealing an inverse temporal relationship with iPTH trends. These comprehensive biochemical profiles are detailed in Table 7; Fig. 4.

## Discussion

Our two-stage prospective cohort study demonstrates that the current local nutritional protocol for preterm infants is associated with a high prevalence of phosphate deficiency, leading to significant MBDP risk. We found that this insufficiency, stemming from suboptimal mineral intake, triggers a cascade of biochemical abnormalities and adaptive hormonal responses, particularly in the most premature infants.

We identified inadequate phosphate intake as the core metabolic disturbance in our cohort. This directly manifested as hypophosphatemia and impaired bone mineralization, which we observed as a progressive and sustained rise in ALP, consistently exceeding the physiological threshold of 500 U/L in EPIs and VPIs [10, 15]. The consequent release of calcium from bone, coupled with its inability to be utilized for formation, led to hypercalciuria, with median uCa levels persistently exceeding the 1.0 mmol/L threshold [11]. Furthermore, the failure of sP and sCa levels in EPIs and VPIs to recover to the high normal range typical of their postmenstrual age at birth underscores the unique challenge these infants face in transitioning from passive placental mineral transfer to active postnatal intestinal absorption and renal conservation, a process heavily dependent on nutritional intake and hormonal maturity which our current protocol appears insufficient to support. Together, these findings reflect a persistent mineral debt accrued during the critical early postnatal period.

We documented several key physiological responses to this mineral deficit. The elevated iPTH levels reflect a combined physiological adaptation and a sustained pathological response to chronically low mineral availability. The high level of iPTH strongly suggests a calcium deficiency [1]. The persistence of markedly elevated iPTH indicates a continued physiological stress driven by insufficient intake. However, we observed a paradoxical hypophosphaturia alongside high iPTH, which we attribute to the immature kidney’s reduced sensitivity to iPTH, particularly in EPIs and VPIs, leading to maximized renal phosphate conservation (high TRP). The observed weak correlations between uP and serum levels of both sP and sCa are particularly noteworthy. Under normal physiological conditions, a stronger relationship might be expected. The weakness of these associations in our cohort likely reflects the dominant physiological drive for renal phosphate conservation in a state of severe deficiency. In this context, the kidney’s imperative to minimize phosphate loss overrides and thus attenuates the standard correlation with serum levels. Our finding that Tp/GFR was GA-dependent further confirms the critical role of gradual renal maturation in phosphate handling [16–19]. In contrast to dynamic iPTH, we found that PTHrP decreased from cord blood to the postnatal phase, while FGF23 levels remained elevated.

Our multivariate regression models identified several independent risk factors for MBDP: lower GA, reduced BW, prolonged PN dependence, delayed vitamin D_3_ supplementation (> 14 days), and neonatal comorbidities. We highlight delayed vitamin D_3_ initiation as a key modifiable risk factor. Conversely, preterm formula feeding emerged as a significant protective factor [20–22], underscoring that the pathophysiological cornerstone of MBDP in our cohort was correctable Ca-P deficiency.

Our data support two key clinical actions. First, we must revise local Ca-P and vitamin D_3_ provision protocols to prioritize earlier and more adequate phosphate delivery. This is supported by the work of Hair et al., which demonstrated that delayed introduction of parenteral phosphate is associated with hypercalcemia and subsequent MBDP in EPIs, underscoring the importance of early supplementation to improve later calcium and phosphate homeostasis [23]. Second, we should implement standardized biochemical monitoring—including serial sP, sCa, uP, uCa, and iPTH—to guide individualized supplementation. In conclusion, this study provides a comprehensive pathophysiological basis for MBDP, directly linking suboptimal nutrition to biochemical and hormonal dysregulation. Prioritizing early and adequate phosphate delivery, coupled with proactive monitoring, are essential strategies to improve bone health in this vulnerable population.

Our study has several limitations. First, unmeasured confounders inherent to clinical settings may have influenced phosphate metabolism. Second, sample size constraints limited the granularity of some analyses. Third, a lack of data from the first 72 postnatal hours represents a gap in understanding the immediate transition. Finally, a limitation of our study, and the field in general, is the absence of established reference ranges for hormones regulating phosphate metabolism like iPTH and FGF23 in preterm infants of different GAs. The values we report are specific to our assay and cohort. Therefore, our interpretation focuses on the dynamic patterns and physiological correlations of these hormones rather than on absolute values against a non-existent standard. Future multi-center studies with standardized assays are needed to define these critical reference intervals.

## Conclusions

Our study provides clear evidence that the current local nutritional protocol results in suboptimal calcium and phosphate intake, which directly contributes to a high prevalence of biochemical phosphate deficiency and an elevated risk of MBDP in EPIs and VPIs. We identified delayed vitamin D supplementation and prolonged PN dependence as key modifiable risk factors. Based on these findings, we conclude that revising nutritional protocols to ensure adequate mineral supply—including optimizing mineral content in both peripheral and central lines— and implementing a standardized monitoring panel (including sP, uP, uCa, and iPTH) are critical clinical strategies. This approach enables the early detection of phosphate imbalance and to guide timely supplementation. The dynamic patterns of hormones regulating phosphate metabolism observed in our cohort underscore the complexity of postnatal mineral adaptation; therefore, future large-scale studies are warranted to establish reference ranges and definitively evaluate the utility of these hormones in guiding and monitoring targeted nutritional interventions.

## Data Availability

The datasets generated and/or analysed during the current study are available in the Mendeley Data repository, V1, 10.17632/6m32bh6rjt.1.

## Abbreviations

ALP: Alkaline phosphatase
ANOVA: Analysis of Variance
BW: Birth weight
BPD: Bronchopulmonary dysplasia
Ca-P: Calcium-phosphate
Ca: P: ratio of calcium to phosphate
CI: Confidence interval
EN: Enteral nutrition
EPI: Extremely preterm infants
ESPGHAN: European Society of Paediatric Gastroenterology, Hepatology and Nutrition
ELISA: Enzyme-linked immunosorbent assay
ELBW: Extremely low birth weight
FGF23: Fibroblast growth factor-23
GA: Gestational age
iPTH: Intact parathyroid hormone
IQR: Interquartile range
LPI: Late preterm infants
LBW: Low birth weight
LSD: Least Significant Difference
LR: Likelihood ratio
MBDP: Metabolic bone disease of prematurity
MPI: Moderate preterm infants
NICU: Neonatal intensive care unit
NRDS: Neonatal respiratory distress syndrome
NEC: Necrotizing enterocolitis
1,25(OH)2D: 1,25-Dihydroxyvitamin D
OR: Odds ratio
PTHrP: Parathyroid hormone-related protein
PN: Parenteral nutrition
PICC: Peripherally inserted central catheter
sP: Serum phosphate
sCa: Serum calcium
SD: Standard deviation
sCr: Serum creatinine
SGA: Small for gestational age
25(OH)D: 25-Hydroxyvitamin D
TRP: Tubular reabsorption of phosphate
Tp/GFR: Renal phosphate threshold concentrations
uP: Urine phosphate
uCa: Urine calcium
uCr: Urine creatinine
VPI: Very preterm infants
